# Pulmonary vein isolation does not alter cardiovascular afferent autonomic reflexes in atrial fibrillation

**DOI:** 10.1002/joa3.70119

**Published:** 2025-06-23

**Authors:** Varun Malik, Adrian D. Elliott, Gijo Thomas, Bradley Pitman, John L. Fitzgerald, Glenn D. Young, Leonard F. Arnolda, Dennis H. Lau, Prashanthan Sanders

**Affiliations:** ^1^ Centre for Heart Rhythm Disorders University of Adelaide and Royal Adelaide Hospital Adelaide South Australia Australia

**Keywords:** atrial fibrillation, autonomic nervous system, isometric handgrip reflex, lower body negative pressure, Valsalva reflex

## Abstract

**Background:**

Pulmonary vein isolation (PVI) remains the cornerstone of atrial fibrillation (AF) ablation. We previously demonstrated abnormal cardiac volume‐sensitive reflexes (whose receptors are co‐located in veno‐atrial tissue) in AF patients. Whether PVI disrupts afferent nerves is unknown.

**Objectives:**

Evaluate whether PVI disrupts afferent volume‐sensitive reflexes.

**Methods:**

We consecutively studied autonomic reflexes in AF patients undergoing PVI, repeating the study post‐PVI, if AF‐free >6 months. We excluded patients with AF recurrence/procedural complications, allowing repeat procedures. We measured beat‐to‐beat mean arterial pressure (MAP) and heart rate (HR) continuously during low‐level Lower Body Negative Pressure (LBNP), at 0, −20 and −40 mmHg (predominantly testing volume baroreceptors); Valsalva reflex (predominantly arterial baroreceptors); and Isometric Handgrip reflex (IHR, both). LBNP produces reflex vasoconstriction, evaluated from forearm blood flow (FBF ∝ 1/vascular resistance).

**Results:**

18 patients were studied pre‐PVI; *n* = 9 completed both visits. Mean age was 64 ± 3 years (78% male); BMI 28 ± 1 kg/m^2^; LA size 37 ± 2 mL/m^2^; and left ventricular function 65 ± 3%. Despite alterations in heart rate variability (HRV), there was no difference in IHR, Valsalva, or LBNP responses pre‐ versus post‐PVI. During LBNP, MAP decreased slightly both pre‐ (−1.6 ± 3%) and post‐PVI (−2.8 ± 1.8%); *p* = .7. HR increased similarly (*p* = .7) pre‐ (10.6 ± 6.4%) and post‐PVI (7.2 ± 1.5%). FBF response was unchanged (*p* = .8). Resting (arterial) baroreflex sensitivity was unaltered.

**Conclusion:**

PVI does not impair cardiovascular reflexes involving afferent baroreceptors, suggesting HRV changes reflect efferent modulation or ablation adequacy rather than afferent disruption. Whether disrupting sino‐atrial efferent nerves represents a marker of adequate ablation or influences PVI outcomes requires evaluation.

## Graphical abstract

Successful (radiofrequency) PVI has no effect on cardiovascular afferent autonomic reflex integrity, despite alterations in heart rate variability (HRV). Therefore, are observations of HRV alterations being predictive of PVI outcomes simply a marker of adequate ablation or does the autonomic nervous system directly influence PVI outcomes?

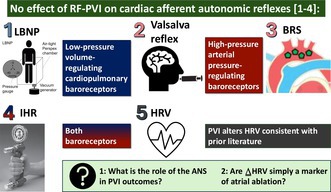

## 1 INTRODUCTION

Pulmonary vein isolation (PVI) is a keystone therapeutic option to maintain sinus rhythm (SR) in patients with atrial fibrillation (AF).^1^ PVI inadvertently disrupts left atrial autonomic ganglia and innervation, which is well known to alter cardiac autonomic efferent tone, as measured by alterations in heart rate and heart rate variability (HRV).^2^, ^3^, ^4^, ^5^, ^6^, ^7^, ^8^, ^9^, ^10^, ^11^ Conversely, autonomic ganglionic stimulation results in vagally induced heart rate slowing and converts atrial ectopic firing in the pulmonary vein into AF.^12^ Procedures targeting left atrial autonomic ganglia *in addition* to PVI possibly have superior outcomes to PVI alone.^2^, ^7^, ^10^, ^13^, ^14^ Recently, a sub‐analysis of the CIRCA‐DOSE study^11^ demonstrated that PVI, with any method of *thermal* energy (cryotherapy or radiofrequency), altered HRV (possibly disrupting autonomic ganglia), correlating with procedural success.

Although there is precedent for autonomic destruction from PVI and its possible influence on successful outcomes (either directly attributable or as a marker of effective ablation lesions), there are no data describing the effect of PVI on afferent or regulatory autonomic nerves (receptors) found in pulmonary vein‐left atrial (PV‐LA) junctions, particularly those that respond to changes in cardiac volume (volume‐regulating baroreceptors).^15^, ^16^, ^17^, ^18^, ^19^

Here, we evaluate whether PVI disrupts the volume‐regulating reflexes. We performed an array of clinical autonomic reflex tests^18^ in AF patients during SR, scheduled to undergo PVI. To test the effect of PVI, we repeated autonomic reflex tests at least 6 months after clinically successful PVI.

## 2 METHODS

### 2.1 Study population

The study comprised of consecutively enrolled patients, referred to a cardiac electrophysiologist at the Centre for Heart Rhythm Disorders with paroxysmal or persistent AF, selected to undergo PVI. The following were exclusions: amiodarone (preceding 6 months); active malignancy; symptomatic coronary artery disease; significant valvular disease; neurological disorders (Parkinson's disease, autonomic disorders, neuropathy); prior ablations; other arrhythmias; inability to enter a lower body chamber (frail or >120 kg); or inability to withhold anti‐arrhythmic/anti‐hypertensives prior to autonomic testing.

Those who participated in the first visit (pre‐PVI) were re‐contacted for a post‐PVI visit provided they met additional prespecified criteria:

1. Absence of procedural complications (including significant pericardial effusion requiring drainage, pericarditis, or stroke).
2. Patients needed to satisfy full clinical follow‐up at 1 week, 1, 3, and 6 months post‐PVI, including a 12‐lead electrocardiogram per visit and 7‐day Holter monitoring at 3 and 6 months and needed to be symptom‐free, without documented electrocardiographic evidence of AF for at least 6 months post‐PVI.
3. Repeat procedures in the cohort were allowed, provided that at the post‐PVI visit, patients were AF‐free for >6 months.

All participants provided written informed consent, and the study was approved by the University of Adelaide human research ethics committee. This study was prospectively registered with the Australian New Zealand Clinical Trials Registry (ACTRN12619000186156).

### 2.2 Patient preparation

All patients withheld alcohol (24 h) and caffeine (48 h), refraining from vigorous exercise (48 h) prior to the study. All anti‐arrhythmic (rate and rhythm controlling) and anti‐hypertensive medications were withheld for 5 half‐lives. Patients fasted for 4 h, allowing water ad libitum.

Autonomic testing was performed in a climate‐controlled facility (22°C). We collected baseline demographics; risk factors; and echocardiographic cardiac chamber measurements. Post‐PVI visits were conducted in an identical manner to pre‐PVI visits.

### 2.3 Autonomic testing protocol

The autonomic testing protocol in this study was identical to our prior work, where each reflex and its componentry is described in detail.^18^ Briefly, we performed three autonomic tests that interrogate cardiovascular afferent autonomic function: submaximal isometric handgrip reflex (IHR), Valsalva reflex, and Lower Body Negative Pressure (LBNP). While the Valsalva reflex predominantly interrogates high‐pressure arterial baroreceptors, low‐level LBNP (where arterial pressure does not decrease) predominantly assesses low‐pressure (volume‐regulating) cardiopulmonary baroreceptors (in predominantly veno‐atrial locations, including the PV‐LA).^15^ LBNP elicits decreases in cardiac volume, resulting in reflex vasoconstriction.^17^, ^18^ IHR addresses both baroreceptor types.^18^ Hemodynamic parameters, systolic blood pressure (SBP), diastolic pressure (DBP), mean arterial pressure (MAP), and heart rate (HR) were collected continuously for all the three tests using finger photoplethysmography (Human NIBP Nano; ADInstruments, Australia).

We utilized LabChart 8 and PowerLab (ADInstruments, Australia) to analyze standard measures of heart rate variability (HRV) as well as Baroreflex sensitivity (BrS) in SR at baseline. HRV was calculated from <10‐min (minimum 5‐min) recordings. BrS was derived using the sequence method.^20^, ^21^, ^22^, ^23^, ^24^ 5 sequences (minimum of 4 sequences per patient, per visit) consisting of a minimum of 4 consecutively measured heart rate *and* SBP beats (that were either concordantly positive or negative spontaneous changes) were collected using an automated, custom‐made macro developed by ADInstruments (Australia). The derivative of the steepest point of the upstroke of the pulse was used for measurement of each beat. Owing to the short (5–10‐min) epochs collected, as per prior recommendations,^25^, ^26^ we restricted our analysis to the following time domain parameters: heart rate (HR); standard deviation of RR interval (SDRR); proportion (%) of the number of RR intervals >50 ms/total number of RR intervals (pRR50); the square root of the mean squared differences of successive RR intervals (RMSSD); related standard deviation of successive RR interval differences (SDSD) and frequency domain parameters: very low frequency (VLF); low frequency (LF); high frequency (HF). This was done to minimize any limitations from the usage of HRV parameters not suitable for the length of recordings analyzed in this study. Additional LBNP parameters collected to test reflex vasoconstriction were forearm blood flow (FBF) and derived forearm vascular resistance (FVC), using venous occlusion plethysmography, as per our prior studies^17^, ^18^; analyses were blinded as to the level of LBNP and the group.

### 2.4 Pulmonary vein isolation

In this study, PVI was standardized. No specific attempts were made to ablate areas associated with cardiac ganglionated plexi. PVI was performed in the fasting state, under general anesthesia on uninterrupted oral anticoagulation. Three electrode catheters were introduced percutaneously via the right femoral vein under ultrasound guidance. A 7F decapolar steerable catheter (Livewire, Abbott medical, USA) was placed in the coronary sinus and used for pacing and recording. Geometry and voltage maps were created of the left atrium using either a variable size circumferential decapolar mapping catheter (Lasso, Biosense‐Webster, USA) or a high‐density mapping catheter (HD‐Grid Advisor, Abbott medical, USA) during coronary sinus pacing. Maps were created with the EnSite Precision 3‐D mapping system (Abbott medical, USA). An 8F, 4 mm irrigated tip ablation catheter (Flexability D‐F curve, Abbott medical, USA) was used for ablation. They were introduced to the left atrium via trans‐septal puncture (BRK‐1 needle, Abbott medical, USA), and an 8.5F SL‐0 sheath (Abbott medical, USA) for the mapping catheters and, over‐the‐wire, using a steerable sheath (8.5F Agilis, Abbott medical, USA) for the ablation catheter. Trans‐septal puncture was performed with transesophageal echocardiogram guidance, pressure monitoring, and contrast fluoroscopy. A single ring of antral ablation surrounding the pulmonary veins (PV) and posterior wall (PW), en bloc, was created. Bidirectional block (entrance and exit block) and, if present, dissociated firing were ablation endpoints. All veins were re‐checked after an observation period and further ablation was performed if acute conduction recovery was detected to ensure durable isolation of the PVs and posterior wall.

### 2.5 Statistical analysis

Continuous parameters were expressed as mean ± SEM. Categorical variables were expressed as frequencies and percentages and compared using Fisher's exact test. Normality was checked, and data were compared using the Student's *t*‐test or its non‐parametric alternate. Hemodynamic data for each autonomic reflex test were expressed as a percentage change from baseline, and then, the data between time points (pre vs. post) were compared using paired t‐tests (normally distributed data) or Wilcoxon signed rank test (non‐normally distributed data) as appropriate. Statistical analysis was performed using STATA 16 (StataCorp. 2019. Stata Statistical Software: Release 16. College Station, TX: StataCorp LLC) and GraphPad Prism (version 9.02, California, USA). Statistical significance was set at *p* < .05.

## 3 RESULTS

**FIGURE 1:**
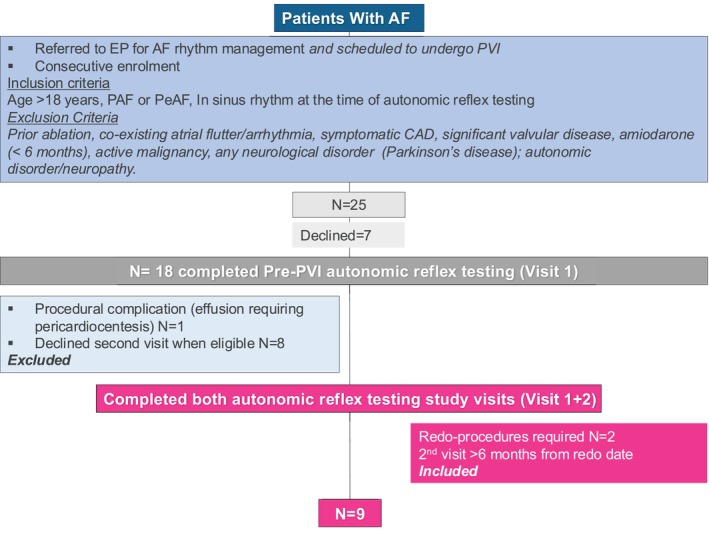
CONSORT Diagram of patient recruitment and establishment of a cohort with completed pre‐ and post‐PVI visits (Visit 1 + 2), where PVI resulted in freedom from AF >6 months.

18 patients were consecutively enrolled pre‐PVI. From these, a cohort of 9 patients was eligible and consented to both study visits. Figure 1: CONSORT diagram. The mean age of the cohort was 64 ± 3 years (78% male); BMI 28 ± 1 kg/m^2^; LA size 37 ± 2 mL/m^2^; and left ventricular ejection fraction 65 ± 3%. Table 1. A redo procedure for recurrence was performed in *n* = 2, and the second study visit was performed if >6 months AF‐free after the last procedure. PV/PW bidirectional block was confirmed in all. Additional lesions (at the operator's discretion) were a mitral isthmus line and ablation of anterior atrial low‐amplitude, fractionated signals (scar) in (*n* = 1). Median duration between study visits was 258 days (range: 184–440 days).

**TABLE 1:** Baseline characteristics.

Baseline characteristics	PVI cohort, *n* = 9
Age	63 ± 2
Males; *n* (%)	7 (78)
Body Mass Index (kg/m^2^)	28 ± 1
Resting MAP	90 ± 4
Resting HR	61 ± 3
LVEF (%)	65 ± 3
LAVI (mL/m^2^)	37 ± 2
Redo due to recurrence during study	2 (22)
*Risk factors*	
Hypertension, *n* (%)	5 (56)
Dyslipidemia	1 (11)
Vascular disease	1 (11)
OSA	3 (33)
Alcohol excess	3 (33)
*Medications*	
ß‐blockers (except Sotalol), *n* (%)	5 (56)
Sotalol, *n* (%)	2 (22)
Flecainide, *n* (%)	3 (33)
Diltiazem, *n* (%)	1 (11)
Digoxin, *n* (%)	0 (0)
ARB/ACEi	4 (44)
Dihydropyridine CCB, *n* (%)	1 (11)
Thiazide diuretic	1 (11)

Abbreviations: ACEi, angiotensin‐converting enzyme inhibitors; ARB, angiotensin receptor blockers; CCB, calcium channel blockers; HR, heart rate; LAVI, left atrial volume indexed to body surface area; LVEF, left ventricular ejection fraction; MAP, mean arterial pressure.

Symptom and rhythm (12‐lead ECG, 7‐day Holter) follow‐up (1 week, 1, 3, and 6 months) showed no clinical recurrence in 7 of 9 patients. In patients that underwent redo‐ procedures, mapping demonstrated gaps in the previous lines and reconnection (*n* = 2). In both cases, clinical follow‐up visits and ECG monitoring were repeated after their repeat PVI procedure at the same time points as above.

### 3.1 Baseline baroreflex sensitivity (BrS)

In the resting, seated state, MAP was 89 ± 5 mmHg pre‐PVI and 93 ± 4 mmHg post‐PVI (*p* = .3). BrS was 4.6 ± 1.1 ms/mmHg pre‐PVI and 6.5 ± 0.7 ms/mmHg post‐PVI. This was not statistically significant (*p* = .2).

### 3.2 Baseline heart rate variability (HRV)

Baseline mean HR was 61 ± 3 pre‐PVI, increasing to 73 ± 3 post‐PVI (*p* = .04). Pre‐ and post‐PVI HRV is presented in Table 2. Although none of the time domain parameters achieved statistical significance, both SDSD and RMSSD numerically halved post‐PVI (*p* = .06) and pRR50 decreased numerically 11% to 4% (*p* = .09), suggesting a trend. Frequency domain HRV characteristics showed a significant decrease in overall power (*p* = .03) and VLF (0.03). The decreases in LF and HF were not statistically significant (*p* = .06, respectively), but once again, suggested a trend.

**TABLE 2:** HRV parameters.

Heart rate variability: Seated, at rest	Pre‐PVI (*n* = 9)	Post‐PVI (*n* = 9)	*p*
HR	61 ± 3	73 ± 3	.04*
*Time domain HRV parameters*			
SDRR (ms)	45.2 ± 7.1	30 ± 4.7	.09
pRR50 (%)	11.4 ± 4.3	3.91 ± 2.3	.09
RMSSD (ms)	40 ± 3	20.7 ± 3	.06
SDSD (ms)	40 ± 9.5	21 ± 3	.06
Frequency domain parameters			
Total Power (μs^2^)	3045.5 ± 1364.9	893.2 ± 253.5	.03*
VLF (%)	39.6 ± 6.1	49.5 ± 4.2	.6
VLF (μs^2^)	908.3 ± 194.4	451.5 ± 153.8	.03*
LF (%)	28.9 ± 3.4	25.1 ± 4.9	.7
LF (μs^2^)	893.8 ± 417.7	222.9 ± 73.2	.06
HF (%)	29.5 ± 4.8	24 ± 3.4	.1
HF (μs^2^)	1120.1 ± 709.3	211.7 ± 72.8	.06
LF/HF	1.18 ± 0.3	1.4 ± 0.4	.3

Abbreviations: HF, high frequency; HR, heart rate; LF, low frequency; pRR50, proportion (%) of the number of RR intervals >50 ms/total number of RR intervals; RMSSD, the square root of the mean squared differences of successive RR intervals; SDRR, standard deviation of RR interval; SDSD, the related standard deviation of successive RR interval differences; VLF, very low frequency. *p < 0.05.

### 3.3 Submaximal isometric handgrip reflex

**FIGURE 2:**
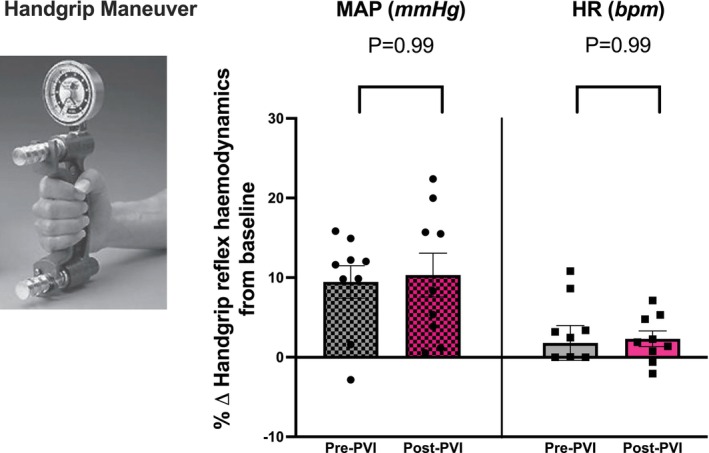
IHR hemodynamic responses were compared to their corresponding baseline value prior to IHR and were expressed as percentage change. Data ± SEM. MAP, Mean Arterial Pressure (mmHg) & HR, Heart rate (bpm). Pre and post comparisons for IHR; *p* < .05.

IHR increased MAP similarly both pre‐PVI and post‐PVI (+9 ± 2% and +10 ± 3%; *p* = .99). HR increased slightly pre‐PVI in response to IHR (+2 ± 2%) and similarly post‐PVI (+2 ± 1%). HR response to IHR was not significantly different between time points (*p* = .99). Figure 2. Raw hemodynamic responses to IHR are presented in Table 3.

**TABLE 3:** Hemodynamic responses to autonomic reflex tests Pre and Post‐PVI.

IHR	Pre PVI (*n* = 9)		Post‐PVI (*n* = 9)		*p* value
	Pre‐IHR	Post‐IHR	Pre‐IHR	Post‐IHR	
MAP	91 ± 6	100 ± 8	94 ± 7	102 ± 6	.99
SBP	137 ± 11	149 ± 12	138 ± 7	151 ± 6	.5
DBP	70 ± 5	75 ± 6	73 ± 6	77 ± 5	.99
HR	58 ± 3	60 ± 4	73 ± 3	75 ± 3	.99

Valsalva					
Valsalva stage II	II_EARLY_	II_LATE_	II_EARLY_	II_LATE_	*p*
MAP	94 ± 8	120 ± 14	94 ± 5	118 ± 10	.6
SBP	122 ± 11	148 ± 17	124 ± 11	130 ± 9	.1
DBP	86 ± 8	105 ± 12	86 ± 5	103 ± 10	.99
HR	73 ± 5	85 ± 5	83 ± 5	91 ± 5	.2

Valsalva stage IV	IV_BASELINE_	IV_OVERSHOOT_	IV_BASELINE_	IV_OVERSHOOT_	*p*
MAP	88 ± 6	125 ± 11	92 ± 6	123 ± 7	.4
SBP	133 ± 10	187 ± 16	136 ± 17	177 ± 9	.1
DBP	68 ± 5	89 ± 9	72 ± 6	90 ± 5	.8
HR	58 ± 3	69 ± 5	72 ± 3	82 ± 3	.7

LBNP (level)	0 mmHg	−20 mmHg	−40 mmHg	0 mmHg	−20 mmHg	‐ 40 mmHg	*p*
MAP	89 ± 5	90 ± 6	90 ± 7	93 ± 5	92 ± 5	91 ± 5	.3
SBP	130 ± 8	128 ± 9	126 ± 10	139 ± 7	135 ± 7	132 ± 7	.8
DBP	69 ± 5	72 ± 4	73 ± 5	71 ± 4	72 ± 5	73 ± 5	.4
HR	58 ± 5	59 ± 4	66 ± 3	67 ± 3	69 ± 3	71 ± 3	.3
FBF	1.55 ± 0.2	1.09 ± 0.2	1.17 ± 0.2	1.87 ± 0.3	1.56 ± 0.2	1.31 ± 0.1	.9
FVC	1.77 ± 0.3	1.24 ± 0.2	1.30 ± 0.3	2.08 ± 0.4	1.73 ± 0.2	1.46 ± 0.2	.99

*Note*: Data ± SEM. Hemodynamic parameters for each autonomic reflex test: MAP, Mean Arterial Pressure (mmHg); SBP, Systolic Blood Pressure (mmHg); DBP, Diastolic Blood Pressure (mmHg); HR, Heart rate (bpm). Additional LBNP venous occlusion plethysmography parameters were also collected: FBF, Forearm Blood Flow (%/min); FVC, Forearm vascular conductance (100*FBF/MAP, arbitrary units). Differences between time points (pre‐ and post‐PVI) for each hemodynamic parameter are represented by P values in the adjacent column; *p* < .05. Valsalva phases were compared to corresponding baseline value prior to Valsalva and were expressed as percentage change. Between group comparisons for Valsalva for each autonomic phase: II_LATE_ – II_EARLY_ or IV_OVERSHOOT_ – IV_BASELINE_ were then compared between time points (Pre vs. Post‐PVI; *p* < .05).

### 3.4 Valsalva reflex

**FIGURE 3:**
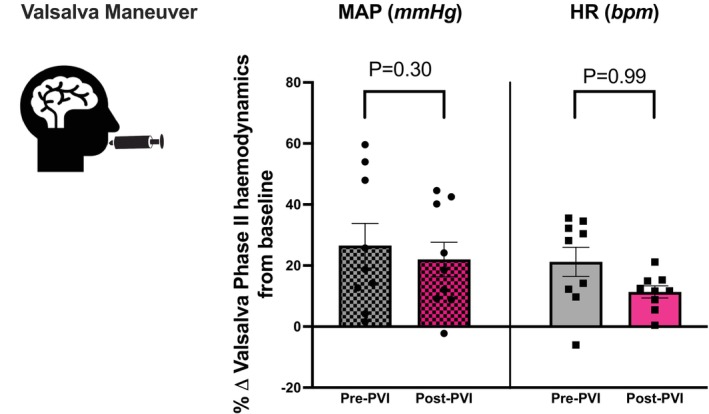
Valsalva phase II response was compared to each corresponding baseline value prior to Valsalva and was expressed as percentage change. Data ± SEM. MAP, Mean Arterial Pressure (mmHg) & HR, Heart rate (bpm). Pre and postcomparisons for Valsalva for Phase II (autonomic phase: II_LATE_ – II_EARLY_; *p* < .05).

There are 4 phases, of which 2 are mechanical. Autonomic phases (II and IV) represent the effect of low blood pressure on sympathetic efferents and a return to baseline function, respectively.^18^ During phase II, pre‐PVI MAP increased by +27 ± 7% and post‐PVI MAP also increased by +22 ± 6% (*p* = .6). HR increased by +21 ± 5% and +11 ± 7% pre‐ and post‐PVI, respectively (*p* = .99). Figure 3. During phase IV, there was an overshoot of both MAP and HR at both time points (*p* = .4 and *p* = .7, respectively). Raw hemodynamic data at both autonomic phases are presented in Table 3.

### 3.5 Lower Body Negative Pressure reflex

**FIGURE 4:**
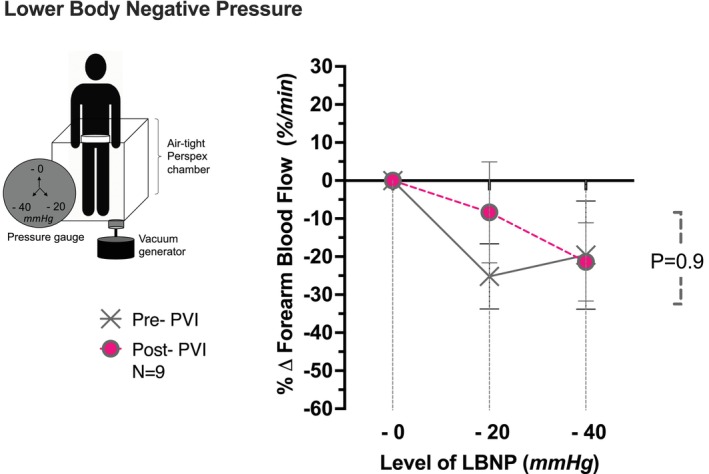
LBNP. Forearm Blood Flow (FBF) response to LBNP—20 mmHg and—40 mmHg negative pressure. Expressed as percentage change from baseline. Data ± SEM. Pre and post CV comparisons; *p* < .05.

During low‐level LBNP, MAP was maintained similarly both pre‐ (+1.0 ± 4%) and post‐PVI (−2.8 ± 2%); *p* = .3. HR increased similarly (*p* = 0.3) pre‐ (+16 ± 7%) and post‐PVI (+7 ± 2%). FBF vasoconstriction response was unchanged (*p* = 0.9); FBF decreased pre‐PVI (−16 ± 18%) and post‐PVI (−21 ± 10%; Figure 4). FVC also decreased pre‐PVI (−18 ± 17%) and post‐PVI (−19 ± 11%; Table 3).

## 4 DISCUSSION

In a cohort of 9 prospective AF patients who underwent comprehensive clinical autonomic testing before and at least 6 months after successful PVI:

1. PVI, from direct thermal injury to PV‐LA tissue which includes autonomic ganglia and receptors regulating cardiac volume, shows no evidence of disruption to volume‐regulating autonomic reflexes (IHR and LBNP). Both Valsalva responses (arterial pressure‐regulating baroreflexes) and resting BrS were intact. HRV showed consistency with prior studies examining autonomic effects of PVI on heart rate.

### 4.1 Central illustration

In the PV‐LA, there is an anatomic co‐location of the AF‐triggering ectopic atrial beats^27^ with (i) autonomic afferent (cardiopulmonary, low‐pressure, volume‐regulating) baroreceptors,^15^, ^16^, ^17^, ^18^ principally responsible for low‐level LBNP reflexes^19^; (ii) intracardiac neurons^28^; and (iii) ganglionated plexi (GP).^29^, ^30^ Each of which is potentially affected by thermal tissue damage (PVI). Importantly, non‐thermal energy (pulsed field ablation), which is gaining widespread adoption, may not impact neural tissue.^31^, ^32^ Therefore, whether there is any direct neural tissue destruction from thermal ablation requires scrutiny first.

Specific autonomic ablation targeting the location of GP, either anatomically,^13^ or through the recognition of the physiologic effects of GP on atrial signals (complex fractionated atrial electrograms),^14^ or the Vagus (sinus bradycardia, atrioventricular block, asystole or hypotension),^2^ remains controversial. When added to PVI, GP ablation may be associated with increased efficacy.^2^, ^7^, ^10^, ^13^, ^14^ Indeed, there is also inadvertent GP damage during standard PVI, which is postulated to contribute to procedural success.^30^ However, the mechanism remains unclear and GP ablation without PVI is not superior to PVI alone.^33^ Further, preclinical studies suggest that GP ablation may, in fact, be pro‐arrhythmic.^34^ Thus, either there is some direct link between such autonomic effects and successful outcomes, or increases in HR and enhanced sympathetic balance on sino‐atrial control (HRV),^2^, ^3^, ^7^, ^10^, ^11^, ^13^ are simply a marker of adequate ablation in these areas to produce bidirectional block (standard end point of PVI). The variability of the duration of HRV changes from PVI date should be emphasized.^2^, ^3^, ^7^, ^11^, ^14^ Also, there are several limitations of HRV in the assessment of autonomic function, as specific perturbations of autonomic tone are not well assessed. In our study, while we have identified similar alterations in HRV following PVI to other studies, we did not identify any other alterations in cardiovascular reflexes.

The mechanism for the initial “vagal” response (bradycardia, atrio‐ventricular block, asystole, or hypotension) during ablation, followed by tachycardia/HRV alteration identified in this study and others^2^, ^3^, ^7^, ^11^, ^14^ is likely from stimulation, then destruction of vagal efferent innervation to the sino‐atrial node. These nerves transit epicardially, surrounding the PV‐LA, converging in ganglia containing parasympathetic postganglionic neurons to the sino‐atrial and atrioventricular nodes.^35^, ^36^ How this could explain the association of HRV with better PVI outcomes is unclear. Seminal extensions^37^ of experiments by Wijffels et al.^38^ did not find efferent autonomic blockade influenced AF maintenance. Alternatively, afferent type B atrial receptors^39^ that respond to changes in blood volume (low‐pressure volume‐regulating cardiopulmonary receptors)^16^, ^19^, ^39^ in the PV‐LA could theoretically be influenced by PVI to explain this.

The current study implies that prior findings of HRV associations with positive outcomes following PVI^2^, ^3^, ^11^ are unlikely mediated by afferent baroreflex remodeling. Neither cardiopulmonary volume‐regulating low‐pressure baroreceptors, nor arterial baroreceptor function (Valsalva reflex and resting baroreflex sensitivity) were affected by PVI. Conversely, the effect of PVI in restoring SR does not appear to influence volume‐regulating baroreflex function.

There are several limitations. Firstly, this was a small, single‐center study. In refining inclusion to successful clinical outcomes (with stringent follow‐up), we mostly eliminated the possibility of insufficient ablation. However, this, together with the time intensive nature of autonomic testing, reduced the sample size. Nevertheless, we clearly demonstrated absence of any disruption of afferent reflexes from PVI. Some of our HRV measures did not achieve statistical significance (small sample size); however, the remaining parameters were significant and consistent with prior studies. Small differences may have been missed; however, the data are adequate to show absence of destruction, especially compared to deficiencies detected during AF.^18^ Medication withdrawal might result in autonomic differences (particularly HRV). Lack of continuous (implantable) monitoring is a limitation. We did not acutely measure autonomic function during PVI due to its difficulty and interpretability under general anesthesia nor did we document vagal effects. We did not study pulsed field ablation, an emerging PVI method, due to limited availability outside specific studies, although recent reports suggest diminished,^31^ or, lack of HRV change, or neural injury following PVI.^32^ Specific atrial remodeling of intracardiac neurons from PVI remains to be investigated. Given the limited sample size, subtle alterations in afferent autonomic function may have gone undetected. Nonetheless, the consistent findings across multiple reflex tests suggest that gross disruption of afferent cardiovascular reflexes by PVI is unlikely (Table 4).

**TABLE 4:** Summary of the effect of PVI on hemodynamic afferent reflexes.

Reflex	Afferent type	Hemodynamic effect	Effect of PVI	
			*p* value	Interpret
LBNP	Low‐pressure volume‐regulating cardiopulmonary baroreceptors	MAP preserved, HR↑	NS	No change
Valsalva	High‐pressure (arterial) blood pressure‐regulating baroreceptors	MAP↑↑ HR ↑	NS	Preserved
IHR	Both baroreceptor types	MAP↑ HR ~	NS	No change

*Note*: Each reflex is listed together with which afferent receptors integrity is tested as well as the hemodynamic effects observed in terms of MAP, mean arterial pressure and HR, heart rate. Differences between the time points (pre vs. post‐PVI) are represented by *p* values in the adjacent column. NS, Non‐significant (*p* > .05). Interpretation of the effect of PVI on the integrity of each reflex tested.

These results are encouraging in that PVI is not associated with depressed autonomic afferent reflexes. Conversely, pre‐existing autonomic dysfunction seen in AF patients (in SR) is not ameliorated by PVI, thus, underlying mechanisms remain elusive. This study adds to the literature, which relies heavily on HRV, despite its inherent limitations and indirectness. Based on this study, the specific role of the autonomic nervous system (ANS) in the success of PVI (predicated on HRV) needs to be reassessed.

## 5 Conclusion

Cardiovascular reflex tests of afferent integrity, particularly those elicited by decreased cardiac volume (cardiopulmonary low‐pressure baroreceptors), that are co‐located in pulmonary vein‐atrial junctions, are not altered by PVI. More work to (i) identify whether intrinsic cardiac ANS remodeling occurs due to PVI and (ii) determine whether these influence clinical outcomes in AF patients undergoing catheter ablation is needed.

### 5.1 Clinical perspectives

1. PVI is not associated with depressed cardiovascular autonomic reflex integrity, particularly those elicited by decreased cardiac volume (cardiopulmonary low‐pressure baroreceptors), that are co‐located in pulmonary vein–atrial junctions.
2. HR and HRV, which evaluate only autonomic influences on the sino‐atrial node, have been used as markers of cardiac autonomic function; however, the specific role that HRV plays on overall autonomic integrity and success of the PVI procedure is unclear.
3. Given the wide uptake of PFA (a non‐thermal technology) and that it appears to have minimal effects on HRV, the value of this marker appears limited and further specific tests of cardiac autonomic function are warranted.

## FUNDING INFORMATION

This study was funded by the Centre for Heart Rhythm Disorders at the University of Adelaide. Dr Fitzgerald is supported by an Australian Postgraduate Award Scholarship from the University of Adelaide. Dr Elliott is supported by a Future Leader Fellowship from the National Heart Foundation of Australia. Dr Lau is supported by a Mid‐Career Fellowship from The Hospital Research Foundation. Dr Sanders is supported by the National Health and Medical Research Council of Australia.

## CONFLICT OF INTEREST STATEMENT

Dr. Dennis H. Lau reports that the University of Adelaide (UoA) has received on his behalf lecture and/or consulting fees from Abbott Medical, Biotronik, Medtronic, and Microport CRM. Dr. Prashanthan Sanders reports having served on the advisory board of Biosense‐Webster, Medtronic, Abbott, Boston Scientific, Pacemate, and CathRx. Dr. Prashanthan Sanders reports that the UoA has received on his behalf lecture and/or consulting fees from Medtronic, Abbott, Boston Scientific, and Pfizer. Dr. Prashanthan Sanders reports that the UoA has received on his behalf research funding from Medtronic, Abbott, Boston Scientific, and Microport. All remaining authors have no conflicts.

## ETHICS STATEMENT

All participants provided written informed consent, and the study was approved by the University of Adelaide human research ethics committee. This study was prospectively registered with the Australian New Zealand Clinical trials registry (ACTRN12619000186156).

## Data Availability

The data are available from the corresponding author, VM, upon reasonable request.
